# Anaplastic Carcinoma of the Pancreas: A Rare Clinical Entity

**DOI:** 10.7759/cureus.1782

**Published:** 2017-10-18

**Authors:** Erkan Oymaci, Savas Yakan, Mehmet Yildirim, Asuman Argon, Ozan Namdaroglu

**Affiliations:** 1 Department of Surgery, University of Health Sciences,izmir Bozyaka Education and Research Hospital,; 2 Department of Pathology, University of Health Sciences,izmir Bozyaka Education and Research Hospital,

**Keywords:** anaplastic carcinoma, pancreas, surgery

## Abstract

Anaplastic carcinoma of the pancreas (ACP) is a very rare histologic subtype of pancreatic cancer and associated with more aggressive and poor prognosis than pancreatic ductal adenocarcinoma. We aimed to review this rare entity and discuss its clinical features, diagnosis and therapy. We presented a case of a 63-year-old male patient that diagnosed as ACP with cyst formation at a tertiary medical center with a detailed review of the current medical literature. We performed pancreaticoduodenectomy operation with lymph node dissection after diagnosis. Any complication after surgery was not observed. Anaplastic pancreas carcinomas are associated with poor survival when compared to invasive ductal adenocarcinomas. Clinical, radiological, laboratory and histological features may be helpful in making differential diagnosis and should be kept in mind in the diagnosis of this rare pancreatic malignancy.

## Introduction

Anaplastic carcinoma of the pancreas is a rare aggressive pancreatic tumor and accounts for 2-7% of all pancreatic cancers with a male predominance [1,2]. It is a rare pancreatic tumor of epithelial origin, frequently showing various morphologies which include spindle cell type, pleomorphic cell type and giant cell type. Many terms have been used for this tumor which implies pleomorphic carcinoma, pleomorphic giant cell carcinoma, pleomorphic large cell carcinoma, sarcomatoid carcinoma and undifferentiated carcinoma [1-3]. According to the guidelines of the World Health Organization (WHO) in 2010, these carcinomas were classified as undifferentiated (anaplastic) carcinomas. We herein present a case of anaplastic carcinoma of the pancreas (ACP) with remarkable intraductal tumor growth into the head of pancreas.

## Case presentation

A 63-year-old male patient was admitted to our hospital due to abdomen and back pain, jaundice and weight loss. Laboratory investigations revealed hemoglobin: 12 g/L, total/direct bilirubin: 2.5/1.9 mg/dl, carcinoembryonic antigen: 8.5 (0-3.4) ng/ml, and carbohydrate antigen (CA) 19-9: 218.7 (<39) U/ml. Transabdominal ultrasonography showed suspected pancreatic mass lesion, and abdominal computed tomography revealed a hypo-echoic mass lesion in the head of the pancreas that accompanied with severe dilatation of the biliary duct. No distant metastasis or lymph node involvement was detected. We performed pancreatoduodenectomy and lymph node dissection.

**Figure 1:**
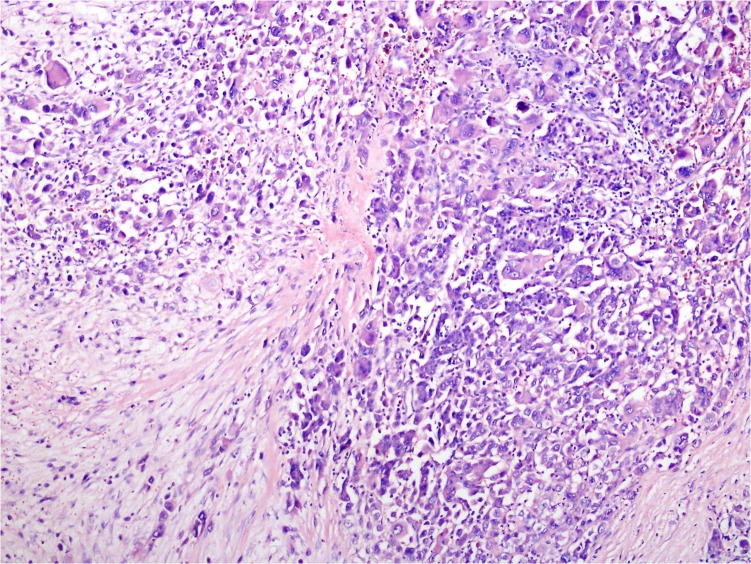
Tumor is composed of poorly cohesive, pleomorphic mononuclear cells and bizarre giant cells. The scanty stroma between the neoplastic cells and necrosis is observed between neoplastic cells (Hematoxylin & Eosin, x10).

On macroscopic examination, the tumor was localized in the head of pancreas and measured 5 x 4 x 3.5 cm. On cross section, it was rubbery and fleshy and also revealed hemorrhage and extensive necrosis. The tumor grossly infiltrated into peripancreatic adipose tissue. Histopathologically the tumor, showed, composed of poorly cohesive, pleomorphic mononuclear cells admixed with bizarre multinucleated giant cells that contain abundant eosinophilic cytoplasm, and scant stroma (Figure 1).

**Figure 2:**
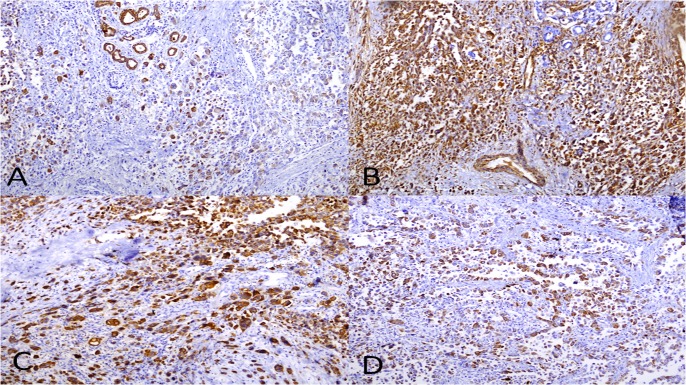
Immunohistochemical staining of tumor. (A) Cytokeratin 7 (x4); (B) Vimentin (x10); (C) CD 68 (x10) and (D) epithelial membrane antigen (EMA) (x10).

Extensive lymphatic and perineural invasions were observed. Immunohistologic studies showed the neoplastic cells were positive with cytokeratin 7, CD 68, epithelial membrane antigen (EMA) and vimentin (Figure 2).

The final pathological diagnosis was anaplastic carcinoma of the pancreas.

The postoperative course was uneventful and the patient discharged from hospital without complication after surgery. The patient died four months after surgery due to systemic adverse effect of the disease.

## Discussion

Anaplastic carcinoma of the pancreas is a very rare histologic subtype of pancreatic cancer and associated with more aggressive tumor and poor prognosis behavior than pancreatic ductal adenocarcinoma. Also, it is frequently diagnosed at an advanced stage with a cystic bulky tumor and adjacent organ involvement. The incidence of anaplastic carcinoma changes between 2.1% and 6.8% among reported case series [4, 5]. It was first reported by Sommers and Meissner as pleomorphic carcinoma. Patients usually have huge tumors showing rapid growth when detected and associated with a very poor prognosis [6]. It is usually seen as a large cystic pancreatic tumor with areas of hemorrhage and necrosis. In our case, the tumor was rubbery and fleshy that revealed hemorrhage and extensive necrosis and grossly infiltrated into peripancreatic adipose tissue. The clinical features caused by this rare tumor are abdominal pain, fatigue, jaundice, body weight loss, anorexia and back pain, which remind symptoms of adenocarcinoma. Hoshimoto, et al. stated that anaplastic carcinoma should be considered when diagnosing pancreatic tumors with a cyst-­like appearance, especially in the presence of severe anemia, elevated leucocyte counts, or elevated serum CA 19­-9 levels [7]. In our case, laboratory examinations revealed anemia and marked elevated CA 19­-9 level (218 U/mL).

ACP is associated with poorer survival when compared to invasive ductal adenocarcinomas. Neither curative resection nor chemotherapy or radiotherapy has been shown to have any benefit due to the aggressive nature and rapid recurrence rates of the disease [8]. Palliative care and close monitoring are the only therapeutic options in most of the cases. Treatment alternatives for this dismal disease remain to be defined.

## Conclusions

In conclusion, ACP is a rare tumor, the pre-operative diagnosis of which can be difficult. In our case, the tumor presented unique features with extensive lymphatic and perineural invasions and this case might help us to better understand the pathogenesis of this entity. Complete surgical excision is the treatment of choice to accurately establish the diagnosis and treatment.
